# 3-(4-Chloro­benzo­yl)-4-(4-chloro­phen­yl)-1-phenethyl­piperidin-4-ol

**DOI:** 10.1107/S1600536811018034

**Published:** 2011-05-20

**Authors:** Abdullah Aydın, Mehmet Akkurt, Ebru Mete, Ertan Sahin, Halise Inci Gul

**Affiliations:** a Department of Science Education, Faculty of Education, Kastamonu University, 37200 Kastamonu, Turkey; b Department of Physics, Faculty of Sciences, Erciyes University, 38039 Kayseri, Turkey; c Department of Chemistry, Faculty of Sciences, Ataturk University, 25240 Erzurum, Turkey; d Department of Pharmaceutical Chemistry, Faculty of Pharmacy, Ataturk University, 25240 Erzurum, Turkey

## Abstract

In the title compound, C_26_H_25_Cl_2_NO_2_, the piperidine ring adopts a chair conformation with a *cis* configuration of the carbonyl and hy­droxy substituents. The dihedral angle between the aromatic rings of the chloro­benzene groups is 24.3 (2)°. The phenyl ring forms dihedral angles of 59.4 (3) and 44.1 (3)° with the benzene rings. In the crystal, mol­ecules are linked by inter­molecular O—H⋯N and C—H⋯O hydrogen bonds and C—H⋯π inter­actions into layers parallel to the *bc* plane.

## Related literature

For the synthesis and biological activity of Mannich bases, see: Dimmock *et al.* (1991 ▶); Dimmock & Kumar (1997) ▶; Gul *et al.* (2001 ▶, 2004 ▶, 2005 ▶); Atwal *et al.* (1969 ▶); Gul (2005 ▶); Erciyas *et al.* (1994 ▶); Porretta *et al.* (1995 ▶); Piscopo *et al.* (1986 ▶); Manavathu *et al.* (1998 ▶); Vashishtha *et al.* (1998 ▶); Canturk *et al.* (2008 ▶); Suleyman *et al.* (2007 ▶); Yogeeswari *et al.* (2005 ▶); Mete *et al.* (2010*a* ▶,*b* ▶) For MOPAC AM1 theoretical full-geometry optimization, see: Dewar *et al.* (1985 ▶); Stewart (1993 ▶). For bond-length data, see: Allen *et al.* (1987 ▶). For puckering parameters, see: Cremer & Pople (1975 ▶).
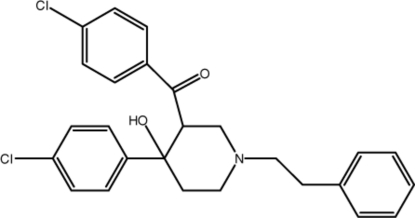


## Experimental

### 

#### Crystal data

C_26_H_25_Cl_2_NO_2_
*M* *_r_* = 454.37
Monoclinic, 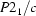

*a* = 16.950 (4) Å
*b* = 12.863 (3) Å
*c* = 10.792 (2) Å
β = 97.779 (13)°
*V* = 2331.3 (9) Å^3^
*Z* = 4
Mo *K*α radiation
μ = 0.30 mm^−1^
*T* = 294 K
0.23 × 0.14 × 0.12 mm

#### Data collection

Rigaku R-AXIS RAPID-S diffractometer
Absorption correction: multi-scan [*XABS2* (Parkin *et al.*, 1995 ▶); cubic fit to sin(θ)/λ, 24 parameters] *T*_min_ = 0.934, *T*_max_ = 0.965
4830 measured reflections
4830 independent reflections
1939 reflections with *I* > 2σ(*I*)

#### Refinement

*R*[*F* ^2^ > 2σ(*F* ^2^)] = 0.097
*wR*(*F* ^2^) = 0.148
*S* = 1.07
4830 reflections
282 parameters
H-atom parameters constrained
Δρ_max_ = 0.18 e Å^−3^
Δρ_min_ = −0.13 e Å^−3^

### 

Data collection: *CrystalClear* (Rigaku/MSC, 2005 ▶); cell refinement: *CrystalClear*; data reduction: *CrystalClear*; program(s) used to solve structure: *SIR97* (Altomare *et al.*, 1999 ▶); program(s) used to refine structure: *SHELXL97* (Sheldrick, 2008 ▶); molecular graphics: *ORTEP-3 for Windows* (Farrugia, 1997 ▶) and *PLATON* (Spek, 2009 ▶); software used to prepare material for publication: *WinGX* (Farrugia, 1999 ▶).

## Supplementary Material

Crystal structure: contains datablocks global, I. DOI: 10.1107/S1600536811018034/rz2590sup1.cif

Structure factors: contains datablocks I. DOI: 10.1107/S1600536811018034/rz2590Isup2.hkl

Supplementary material file. DOI: 10.1107/S1600536811018034/rz2590Isup3.cml

Additional supplementary materials:  crystallographic information; 3D view; checkCIF report

## Figures and Tables

**Table 1:** Hydrogen-bond geometry (Å, °) *Cg*2 and *Cg*3 are the centroids of the C15–C20 and C21–C26 benzene rings, respectively.

*D*—H⋯*A*	*D*—H	H⋯*A*	*D*⋯*A*	*D*—H⋯*A*
O1—H1*A*⋯N1^i^	0.82	2.11	2.879 (5)	155
C13—H13*B*⋯O2^ii^	0.97	2.54	3.372 (5)	144
C2—H2⋯*Cg*2^iii^	0.93	2.85	3.739 (9)	159
C16—H16⋯*Cg*3^iv^	0.93	2.85	3.646 (5)	144

Symmetry codes: (i) 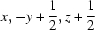; (ii) 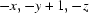; (iii) 
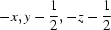
; (iv) 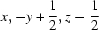.

**Fig. 1.**
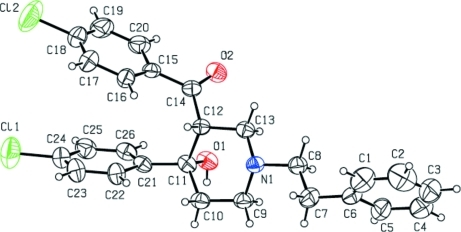
The molecular structure of the title compound with displacement ellipsoids drawn at the 30% probability level.

**Fig. 2.**
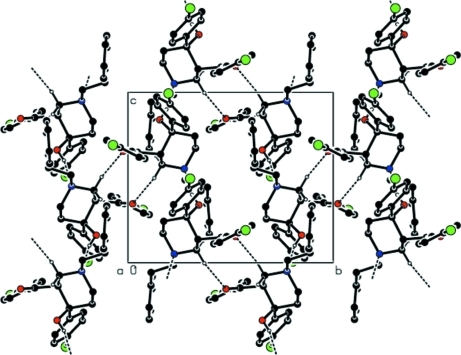
Crystal packing of the title compound viewed down the a axis. H atoms not involved in hydrogen bonds (dashed lines) are omitted for clarity.

**Fig. 3.**
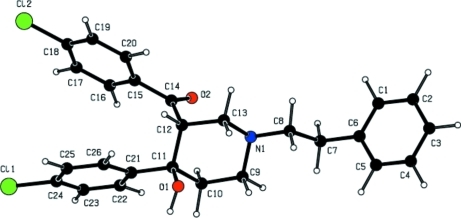
A spatial view of the calculated molecule of the title compound.

## supplementary crystallographic information

### Comment

The title compound, C_26_H_25_Cl_2_NO_2_, is a semicyclic mono Mannich base. Mannich bases are generally formed by the reaction between a compound containing reactive hydrogen atom, formaldehyde, and a secondary amine. On occasion, aldehydes other than formaldehyde may be employed, and the secondary amine may be replaced by ammonia and primary amines. The process whereby these compounds are formed is known as the Mannich reaction (Dimmock & Kumar, 1997).

Mannich bases have several biological activities such as antimicrobial (Gul *et al.*, 2001, Gul, 2005; Erciyas *et al.*, 1994; Porretta *et al.*, 1995; Piscopo *et al.*, 1986; Manavathu *et al.*, 1998; Vashishtha *et al.*, 1998), analgesic (Atwal *et al.*, 1969), anti-inflammatory (Gul, 2005; Suleyman *et al.*, 2007) and anticonvulsant activities (Gul *et al.*, 2004; Dimmock *et al.*, 1991). Considerable anticancer activity was also attributed to Mannich bases (Dimmock & Kumar, 1997). It has been reported that these compounds have an inhibiting effect on DNA topoisomerase I (Canturk *et al.*, 2008) and II (Yogeeswari *et al.*, 2005).

The biological activities of Mannich bases were attributed to the thiol alkylation of *α,β*-unsaturated ketones produced from Mannich bases. Mannich bases which have at least one activated hydrogen atom at the β-position of amine can undergo deamination under simulated physiological condition *in vitro* or *in vivo* condition to produce *α,β*-unsaturated ketones which are biologically active species (Gul *et al.*, 2005).

The title compound was tested against seven types of plant pathogenic fungi and three types of human pathogenic fungi using the agar dilution assay (Mete *et al.*, 2010*b*). Cytotoxic activity of the title compound against androgen-independent prostate cancer cells (PC-3) and the biological activity on DNA topoisomerase I enzyme were also reported (Mete *et al.*, 2010*a*).

The molecular structure of the title compound, (I), is shown in Fig. 1. Bond lengths (Allen *et al.*, 1987) and angles are in normal ranges. The piperidine ring (N1/C9–C13) adopts a chair conformation [puckering parameters are *Q*_T_ = 0.590 (4) Å, θ = 4.4 (4) °, φ = 289 (5) ° (Cremer & Pople, 1975)], with atoms C9, C10, C12 and C13 occupying coplanar positions and atoms C11 and N1 on opposite sides of the plane. The carbonyl and hydroxy groups are *cis* configured. The C15–C20 and C21–C26 benzene rings form a dihedral angle of 24.3 (2)° with each other. The C1–C6 phenyl ring forms dihedral angles of 59.4 (3) and 44.1 (3)° with the C15–C20 and C21–C26 benzene rings, respectively. The crystal structure is stabilized by intermolecular O—H···N and C—H···O hydrogen bonds (Table 1, Fig. 2) and C—H···π interactions (Table 1), forming layer parallel to the *bc* plane.

We applied a semiempirical calculation AM1 of (I) with MOPAC (Dewar *et al.*, 1985; Stewart, 1993). Figure 3 shows the conformation of the calculated molecule. The values of the structural parameters of (I) obtained by the results of the theoretical calculations (based on isolated molecules) and X-ray structural determinations in the solid state are almost identical within experimental error. The dihedral angles between the mean planes of the aromatic rings in (I) are listed in Table 2 for comparision. The calculated dipole moment of (I) is 2.119 D. The HOMO and LUMO energy levels are -9.22109 and -0.56402 eV, respectively. We may state that the theoretical calculation of (I) supports the suggestion that the present intermolecular interactions in (I) influence crystal packing.

### Experimental

4'-Chloroacetophenone (10.00 g), paraformaldehyde (1.95 g) and phenylethylamine hydrochloride (5.12 g) in the molar ratio 2:2:1 were stirred and heated in an oil bath. When the temperature reached 365 K, the solid mixture started to melt. When heating continued, the reaction content solidified again totally. The reaction flask was quickly removed from the oil bath. The temperature of the reaction medium spontaneously increased to 377 K. Following the increase in temperature and removal of the flask from the oil bath, ethyl acetate (20 ml) was added to the reaction flask when the temperature had dropped to 338 K. Stirring was continued for 24 h. The formed precipitate was separated by filtration and crystallized from ethanol to obtain 1-(4-chlorophenyl)-3-phenethylamino-1-propanone hydrochloride. The ethyl acetate present in the reaction flask was removed under reduced pressure to obtain the title compound (I) as a viscous orange oil. The compound was purified by column chromatography using a basic Al_2_O_3_ column with ethyl acetate/hexane (1:9 *v*/*v*) as eluent (yield 18%; m. p. 405–407 K). Crystals suitable for X-ray analysis were obtained by slow evaporation of a methanol solution. ^1^H-NMR(DMSO) δ: 1.55 (br d, 1H, *J* = 13.6 Hz), 2.04–2.11 (m, 1H), 2.56–2.91 (m, 8H), 4.30 (dd, 1H, *J* = 11.0, 3.7 Hz), 4.96 (d, OH, *J* = 1.1 Hz), 7.15 (quasi d,2*H*, J = 8.8 Hz), 7.21–7.28 (m, 5H), 7.43 (quasi d, 2H, J = 8.8 Hz), 7.50 (quasi d, 2H, *J* = 8.4 Hz), 7.74 (quasi d, 2H, *J* = 8.8 Hz). ^13^C-NMR(DMSO) δ: 33.5, 39.6, 48.9, 51.3, 52.3, 60.2, 73.1, 126.5, 127.7, 128.3, 128.9, 129.3, 129.4, 130.7, 131.8, 136.0, 138.9, 141.1, 147.2, 202.5. Elemental analysis: C_26_H_25_Cl_2_NO_2_, Calc.(%) / Found (%): C: 68.72/68.76, H: 5.55/5.43, N: 3.08/3.27 (Mete *et al.*, 2010*b*).

### Refinement

H atoms were positioned geometrically, with O—H = 0.82 Å, C—H = 0.93(aromatic), 0.97(methylene) or 0.98 Å (methine), and refined as riding with *U*_iso_(H) = 1.5*U*_eq_(O) or 1.2*U*_eq_(C). The rather high *R* value (0.0967) is due to the poor quality of the crystal.

### Crystal data

**Table:**

C_26_H_25_Cl_2_NO_2_	*F*(000) = 952
*M**_r_* = 454.37	*D*_x_ = 1.295 Mg m^−^^3^
Monoclinic, *P*2_1_/*c*	Mo *K*α radiation, λ = 0.71073 Å
Hall symbol: -P 2ybc	Cell parameters from 5626 reflections
*a* = 16.950 (4) Å	θ = 2.4–30.5°
*b* = 12.863 (3) Å	µ = 0.30 mm^−^^1^
*c* = 10.792 (2) Å	*T* = 294 K
β = 97.779 (13)°	Block, colourless
*V* = 2331.3 (9) Å^3^	0.23 × 0.14 × 0.12 mm
*Z* = 4	

### Data collection

**Table:**

Rigaku R-AXIS RAPID-S diffractometer	4830 independent reflections
Radiation source: Sealed Tube	1939 reflections with *I* > 2σ(*I*)
Graphite Monochromator	*R*_int_ = 0.000
Detector resolution: 10.00 pixels mm^-1^	θ_max_ = 26.5°, θ_min_ = 2.4°
dtprofit.ref scans	*h* = −21→21
Absorption correction: multi-scan [*XABS2* (Parkin *et al.*, 1995); cubic fit to sin(θ)/λ - 24 parameters]	*k* = 0→16
*T*_min_ = 0.934, *T*_max_ = 0.965	*l* = 0→13
4830 measured reflections	

### Refinement

**Table:**

Refinement on *F*^2^	Primary atom site location: structure-invariant direct methods
Least-squares matrix: full	Secondary atom site location: difference Fourier map
*R*[*F*^2^ > 2σ(*F*^2^)] = 0.097	Hydrogen site location: inferred from neighbouring sites
*wR*(*F*^2^) = 0.148	H-atom parameters constrained
*S* = 1.07	*w* = 1/[σ^2^(*F*_o_^2^) + (0.0094*P*)^2^ + 1.4373*P*] where *P* = (*F*_o_^2^ + 2*F*_c_^2^)/3
4830 reflections	(Δ/σ)_max_ < 0.001
282 parameters	Δρ_max_ = 0.18 e Å^−^^3^
0 restraints	Δρ_min_ = −0.13 e Å^−^^3^

### Special details

**Table:**

Geometry. Bond distances, angles *etc*. have been calculated using the rounded fractional coordinates. All su's are estimated from the variances of the (full) variance-covariance matrix. The cell e.s.d.'s are taken into account in the estimation of distances, angles and torsion angles
Refinement. Refinement on *F*^2^ for ALL reflections except those flagged by the user for potential systematic errors. Weighted *R*-factors *wR* and all goodnesses of fit *S* are based on *F*^2^, conventional *R*-factors *R* are based on *F*, with *F* set to zero for negative *F*^2^. The observed criterion of *F*^2^ > σ(*F*^2^) is used only for calculating -*R*-factor-obs *etc*. and is not relevant to the choice of reflections for refinement. *R*-factors based on *F*^2^ are statistically about twice as large as those based on *F*, and *R*-factors based on ALL data will be even larger.

### Fractional atomic coordinates and isotropic or equivalent isotropic displacement parameters (Å^2^)

**Table:**

	*x*	*y*	*z*	*U*_iso_*/*U*_eq_	
Cl1	0.45921 (10)	0.30278 (16)	0.49332 (19)	0.1484 (10)	
Cl2	0.47453 (11)	0.57277 (17)	0.1905 (2)	0.1803 (13)	
O1	0.0660 (2)	0.3454 (2)	0.3384 (3)	0.0716 (12)	
O2	0.08304 (19)	0.5228 (2)	0.1541 (3)	0.0769 (14)	
N1	−0.0050 (2)	0.2257 (3)	0.0508 (3)	0.0590 (16)	
C1	−0.1997 (4)	0.1029 (5)	−0.2619 (7)	0.112 (3)	
C2	−0.2733 (7)	0.1035 (6)	−0.3331 (8)	0.146 (5)	
C3	−0.3396 (5)	0.1102 (6)	−0.2802 (11)	0.136 (5)	
C4	−0.3367 (5)	0.1168 (5)	−0.1543 (9)	0.128 (4)	
C5	−0.2612 (4)	0.1186 (5)	−0.0817 (6)	0.106 (3)	
C6	−0.1933 (3)	0.1121 (4)	−0.1342 (6)	0.074 (2)	
C7	−0.1130 (3)	0.1128 (4)	−0.0571 (5)	0.089 (3)	
C8	−0.0868 (3)	0.2217 (3)	−0.0146 (4)	0.0683 (17)	
C9	0.0000 (3)	0.1708 (4)	0.1714 (4)	0.0685 (17)	
C10	0.0822 (3)	0.1743 (3)	0.2431 (4)	0.0651 (17)	
C11	0.1144 (3)	0.2856 (3)	0.2674 (4)	0.0601 (17)	
C12	0.1052 (2)	0.3410 (3)	0.1367 (4)	0.0521 (17)	
C13	0.0195 (2)	0.3339 (3)	0.0750 (4)	0.0598 (17)	
C14	0.1317 (3)	0.4537 (4)	0.1482 (4)	0.0581 (17)	
C15	0.2176 (3)	0.4812 (4)	0.1536 (4)	0.0581 (17)	
C16	0.2732 (3)	0.4180 (4)	0.1104 (4)	0.070 (2)	
C17	0.3525 (3)	0.4459 (4)	0.1192 (5)	0.089 (2)	
C18	0.3752 (3)	0.5378 (5)	0.1759 (6)	0.096 (3)	
C19	0.3223 (4)	0.6037 (4)	0.2184 (6)	0.102 (3)	
C20	0.2425 (3)	0.5758 (4)	0.2084 (5)	0.082 (3)	
C21	0.2001 (3)	0.2836 (4)	0.3290 (4)	0.0582 (17)	
C22	0.2562 (3)	0.2166 (4)	0.2909 (4)	0.0733 (19)	
C23	0.3354 (3)	0.2222 (4)	0.3407 (5)	0.087 (3)	
C24	0.3593 (3)	0.2945 (5)	0.4313 (5)	0.084 (2)	
C25	0.3063 (4)	0.3599 (4)	0.4732 (5)	0.085 (3)	
C26	0.2267 (3)	0.3553 (4)	0.4218 (4)	0.0694 (19)	
H1	−0.15410	0.09610	−0.30030	0.1340*	
H1A	0.05700	0.31190	0.39960	0.1070*	
H2	−0.27690	0.09920	−0.41970	0.1750*	
H3	−0.38870	0.11030	−0.33040	0.1630*	
H4	−0.38300	0.12010	−0.11710	0.1540*	
H5	−0.25790	0.12430	0.00480	0.1270*	
H7A	−0.07430	0.08360	−0.10570	0.1070*	
H7B	−0.11450	0.06910	0.01580	0.1070*	
H8A	−0.09080	0.26700	−0.08710	0.0820*	
H8B	−0.12280	0.24810	0.04060	0.0820*	
H9A	−0.01560	0.09880	0.15630	0.0820*	
H9B	−0.03720	0.20210	0.22140	0.0820*	
H10A	0.11810	0.13630	0.19680	0.0780*	
H10B	0.08170	0.13950	0.32260	0.0780*	
H12	0.13890	0.30470	0.08370	0.0620*	
H13A	−0.01500	0.36600	0.12880	0.0720*	
H13B	0.01370	0.37200	−0.00330	0.0720*	
H16	0.25700	0.35460	0.07420	0.0840*	
H17	0.38920	0.40320	0.08740	0.1060*	
H19	0.33930	0.66710	0.25400	0.1220*	
H20	0.20580	0.62000	0.23810	0.0990*	
H22	0.24010	0.16680	0.23040	0.0880*	
H23	0.37230	0.17720	0.31310	0.1040*	
H25	0.32310	0.40760	0.53590	0.1020*	
H26	0.19060	0.40100	0.45010	0.0830*	

### Atomic displacement parameters (Å^2^)

**Table:**

	*U*^11^	*U*^22^	*U*^33^	*U*^12^	*U*^13^	*U*^23^
Cl1	0.0835 (12)	0.1827 (19)	0.1682 (18)	−0.0113 (12)	−0.0226 (11)	−0.0167 (15)
Cl2	0.1017 (14)	0.190 (2)	0.243 (3)	−0.0701 (14)	0.0008 (15)	−0.0200 (18)
O1	0.086 (2)	0.076 (2)	0.058 (2)	0.0178 (19)	0.0290 (18)	0.0100 (17)
O2	0.084 (2)	0.063 (2)	0.085 (3)	0.0156 (19)	0.016 (2)	−0.0046 (19)
N1	0.063 (3)	0.059 (3)	0.056 (2)	−0.004 (2)	0.012 (2)	0.005 (2)
C1	0.121 (6)	0.112 (5)	0.103 (6)	−0.018 (4)	0.016 (5)	−0.025 (4)
C2	0.192 (10)	0.118 (6)	0.113 (7)	−0.012 (7)	−0.033 (8)	−0.029 (5)
C3	0.104 (7)	0.109 (6)	0.184 (10)	−0.020 (5)	−0.020 (7)	0.010 (7)
C4	0.100 (6)	0.117 (6)	0.171 (8)	−0.004 (4)	0.032 (6)	0.036 (6)
C5	0.076 (4)	0.131 (6)	0.112 (5)	−0.012 (4)	0.014 (4)	0.029 (4)
C6	0.077 (4)	0.067 (3)	0.079 (4)	−0.016 (3)	0.014 (4)	0.001 (3)
C7	0.078 (4)	0.074 (4)	0.114 (5)	−0.015 (3)	0.006 (4)	−0.002 (3)
C8	0.070 (3)	0.066 (3)	0.070 (3)	−0.001 (3)	0.013 (3)	0.005 (3)
C9	0.076 (3)	0.065 (3)	0.067 (3)	−0.003 (3)	0.019 (3)	0.014 (3)
C10	0.073 (3)	0.060 (3)	0.064 (3)	0.003 (3)	0.015 (3)	0.010 (3)
C11	0.070 (3)	0.063 (3)	0.049 (3)	0.012 (3)	0.014 (3)	0.002 (3)
C12	0.059 (3)	0.050 (3)	0.049 (3)	0.001 (2)	0.014 (2)	0.000 (2)
C13	0.068 (3)	0.059 (3)	0.054 (3)	0.003 (2)	0.014 (2)	0.002 (2)
C14	0.074 (3)	0.057 (3)	0.045 (3)	−0.003 (3)	0.014 (2)	0.002 (2)
C15	0.069 (3)	0.051 (3)	0.053 (3)	−0.007 (3)	0.004 (3)	0.010 (2)
C16	0.061 (3)	0.073 (4)	0.076 (4)	−0.005 (3)	0.007 (3)	−0.002 (3)
C17	0.073 (4)	0.094 (4)	0.098 (4)	−0.016 (3)	0.010 (3)	−0.004 (4)
C18	0.079 (4)	0.092 (5)	0.109 (5)	−0.025 (4)	−0.014 (4)	0.008 (4)
C19	0.118 (6)	0.068 (4)	0.112 (5)	−0.031 (4)	−0.013 (4)	−0.004 (4)
C20	0.101 (5)	0.061 (4)	0.082 (4)	−0.007 (3)	0.003 (3)	0.004 (3)
C21	0.071 (3)	0.060 (3)	0.044 (3)	0.009 (3)	0.009 (2)	0.006 (2)
C22	0.064 (3)	0.085 (4)	0.070 (3)	0.010 (3)	0.006 (3)	−0.010 (3)
C23	0.071 (4)	0.101 (5)	0.088 (4)	0.018 (3)	0.006 (3)	−0.007 (4)
C24	0.066 (4)	0.091 (4)	0.091 (4)	0.000 (3)	−0.002 (3)	0.008 (4)
C25	0.098 (5)	0.078 (4)	0.075 (4)	0.000 (3)	−0.004 (4)	−0.001 (3)
C26	0.087 (4)	0.063 (3)	0.060 (3)	0.012 (3)	0.017 (3)	0.006 (3)

### Geometric parameters (Å, °)

**Table:**

Cl1—C24	1.738 (5)	C21—C22	1.386 (7)
Cl2—C18	1.729 (6)	C22—C23	1.379 (7)
O1—C11	1.422 (6)	C23—C24	1.370 (8)
O2—C14	1.220 (6)	C24—C25	1.353 (8)
O1—H1A	0.8200	C25—C26	1.389 (8)
N1—C9	1.473 (6)	C1—H1	0.9300
N1—C13	1.466 (5)	C2—H2	0.9300
N1—C8	1.469 (6)	C3—H3	0.9300
C1—C2	1.374 (13)	C4—H4	0.9300
C1—C6	1.373 (10)	C5—H5	0.9300
C2—C3	1.330 (15)	C7—H7A	0.9700
C3—C4	1.356 (15)	C7—H7B	0.9700
C4—C5	1.407 (11)	C8—H8A	0.9700
C5—C6	1.352 (9)	C8—H8B	0.9700
C6—C7	1.496 (8)	C9—H9A	0.9700
C7—C8	1.521 (7)	C9—H9B	0.9700
C9—C10	1.500 (7)	C10—H10A	0.9700
C10—C11	1.542 (6)	C10—H10B	0.9700
C11—C12	1.569 (6)	C12—H12	0.9800
C11—C21	1.514 (7)	C13—H13A	0.9700
C12—C14	1.518 (6)	C13—H13B	0.9700
C12—C13	1.517 (5)	C16—H16	0.9300
C14—C15	1.492 (7)	C17—H17	0.9300
C15—C16	1.373 (7)	C19—H19	0.9300
C15—C20	1.393 (7)	C20—H20	0.9300
C16—C17	1.382 (7)	C22—H22	0.9300
C17—C18	1.362 (8)	C23—H23	0.9300
C18—C19	1.358 (8)	C25—H25	0.9300
C19—C20	1.389 (8)	C26—H26	0.9300
C21—C26	1.391 (7)		
			
C11—O1—H1A	109.00	C3—C2—H2	119.00
C8—N1—C9	110.2 (3)	C2—C3—H3	119.00
C8—N1—C13	110.2 (3)	C4—C3—H3	119.00
C9—N1—C13	108.5 (3)	C3—C4—H4	121.00
C2—C1—C6	120.2 (7)	C5—C4—H4	121.00
C1—C2—C3	121.1 (9)	C4—C5—H5	119.00
C2—C3—C4	121.1 (9)	C6—C5—H5	119.00
C3—C4—C5	117.8 (8)	C6—C7—H7A	109.00
C4—C5—C6	121.8 (7)	C6—C7—H7B	109.00
C1—C6—C5	118.0 (6)	C8—C7—H7A	109.00
C1—C6—C7	120.1 (5)	C8—C7—H7B	109.00
C5—C6—C7	121.9 (6)	H7A—C7—H7B	108.00
C6—C7—C8	112.3 (4)	N1—C8—H8A	109.00
N1—C8—C7	113.3 (4)	N1—C8—H8B	109.00
N1—C9—C10	112.3 (4)	C7—C8—H8A	109.00
C9—C10—C11	113.5 (4)	C7—C8—H8B	109.00
O1—C11—C10	112.2 (4)	H8A—C8—H8B	108.00
O1—C11—C12	104.1 (3)	N1—C9—H9A	109.00
C10—C11—C12	106.2 (3)	N1—C9—H9B	109.00
C10—C11—C21	110.8 (4)	C10—C9—H9A	109.00
C12—C11—C21	112.0 (4)	C10—C9—H9B	109.00
O1—C11—C21	111.2 (4)	H9A—C9—H9B	108.00
C11—C12—C13	109.9 (3)	C9—C10—H10A	109.00
C11—C12—C14	111.6 (3)	C9—C10—H10B	109.00
C13—C12—C14	110.4 (3)	C11—C10—H10A	109.00
N1—C13—C12	111.5 (3)	C11—C10—H10B	109.00
O2—C14—C12	120.4 (4)	H10A—C10—H10B	108.00
C12—C14—C15	120.4 (4)	C11—C12—H12	108.00
O2—C14—C15	119.2 (4)	C13—C12—H12	108.00
C14—C15—C16	123.9 (5)	C14—C12—H12	108.00
C14—C15—C20	117.5 (4)	N1—C13—H13A	109.00
C16—C15—C20	118.6 (5)	N1—C13—H13B	109.00
C15—C16—C17	121.9 (5)	C12—C13—H13A	109.00
C16—C17—C18	118.0 (5)	C12—C13—H13B	109.00
Cl2—C18—C19	119.1 (5)	H13A—C13—H13B	108.00
C17—C18—C19	122.3 (5)	C15—C16—H16	119.00
Cl2—C18—C17	118.6 (4)	C17—C16—H16	119.00
C18—C19—C20	119.5 (5)	C16—C17—H17	121.00
C15—C20—C19	119.6 (5)	C18—C17—H17	121.00
C11—C21—C26	120.2 (4)	C18—C19—H19	120.00
C22—C21—C26	117.2 (5)	C20—C19—H19	120.00
C11—C21—C22	122.4 (4)	C15—C20—H20	120.00
C21—C22—C23	121.5 (5)	C19—C20—H20	120.00
C22—C23—C24	119.5 (5)	C21—C22—H22	119.00
Cl1—C24—C23	119.7 (4)	C23—C22—H22	119.00
Cl1—C24—C25	119.3 (5)	C22—C23—H23	120.00
C23—C24—C25	121.0 (5)	C24—C23—H23	120.00
C24—C25—C26	119.6 (5)	C24—C25—H25	120.00
C21—C26—C25	121.2 (5)	C26—C25—H25	120.00
C2—C1—H1	120.00	C21—C26—H26	119.00
C6—C1—H1	120.00	C25—C26—H26	119.00
C1—C2—H2	120.00		
			
C9—N1—C13—C12	62.1 (4)	C10—C11—C12—C14	177.6 (4)
C9—N1—C8—C7	−67.4 (5)	C14—C12—C13—N1	174.0 (3)
C13—N1—C8—C7	173.0 (4)	C11—C12—C14—O2	−96.0 (5)
C8—N1—C13—C12	−177.2 (3)	C13—C12—C14—O2	26.5 (6)
C13—N1—C9—C10	−58.3 (5)	C13—C12—C14—C15	−154.0 (4)
C8—N1—C9—C10	−179.0 (4)	C11—C12—C14—C15	83.5 (5)
C2—C1—C6—C7	−179.2 (6)	C11—C12—C13—N1	−62.5 (4)
C6—C1—C2—C3	−1.8 (11)	O2—C14—C15—C16	−159.1 (4)
C2—C1—C6—C5	1.9 (9)	O2—C14—C15—C20	22.4 (6)
C1—C2—C3—C4	0.1 (12)	C12—C14—C15—C16	21.5 (6)
C2—C3—C4—C5	1.4 (11)	C12—C14—C15—C20	−157.1 (4)
C3—C4—C5—C6	−1.2 (10)	C14—C15—C16—C17	−178.9 (4)
C4—C5—C6—C1	−0.4 (9)	C20—C15—C16—C17	−0.4 (7)
C4—C5—C6—C7	−179.3 (5)	C14—C15—C20—C19	178.4 (5)
C1—C6—C7—C8	102.4 (6)	C16—C15—C20—C19	−0.2 (7)
C5—C6—C7—C8	−78.7 (6)	C15—C16—C17—C18	1.9 (8)
C6—C7—C8—N1	−174.4 (4)	C16—C17—C18—Cl2	178.8 (4)
N1—C9—C10—C11	56.4 (5)	C16—C17—C18—C19	−2.9 (9)
C9—C10—C11—C12	−52.7 (5)	Cl2—C18—C19—C20	−179.3 (5)
C9—C10—C11—C21	−174.6 (4)	C17—C18—C19—C20	2.4 (10)
C9—C10—C11—O1	60.4 (5)	C18—C19—C20—C15	−0.8 (9)
C10—C11—C12—C13	54.7 (4)	C11—C21—C22—C23	174.2 (4)
O1—C11—C12—C13	−63.8 (4)	C26—C21—C22—C23	−1.5 (7)
O1—C11—C12—C14	59.0 (4)	C11—C21—C26—C25	−175.3 (4)
C21—C11—C12—C14	−61.3 (5)	C22—C21—C26—C25	0.5 (7)
O1—C11—C21—C22	168.5 (4)	C21—C22—C23—C24	1.0 (8)
O1—C11—C21—C26	−16.0 (6)	C22—C23—C24—Cl1	−179.4 (4)
C10—C11—C21—C22	43.0 (6)	C22—C23—C24—C25	0.5 (8)
C10—C11—C21—C26	−141.5 (4)	Cl1—C24—C25—C26	178.5 (4)
C12—C11—C21—C22	−75.4 (5)	C23—C24—C25—C26	−1.4 (8)
C12—C11—C21—C26	100.1 (5)	C24—C25—C26—C21	0.9 (8)
C21—C11—C12—C13	175.9 (3)		

### Hydrogen-bond geometry (Å, °)

**Table:**

Cg2 and Cg3 are the centroids of the C15–C20 and C21–C26 benzene rings, respectively.

**Table:**

*D*—H···*A*	*D*—H	H···*A*	*D*···*A*	*D*—H···*A*
O1—H1A···N1^i^	0.82	2.11	2.879 (5)	155
C13—H13B···O2^ii^	0.97	2.54	3.372 (5)	144
C2—H2···Cg2^iii^	0.93	2.85	3.739 (9)	159
C16—H16···Cg3^iv^	0.93	2.85	3.646 (5)	144

Symmetry codes: (i) *x*, −*y*+1/2, *z*+1/2; (ii) −*x*, −*y*+1, −*z*; (iii) −*x*, *y*−1/2, −*z*−1/2; (iv) *x*, −*y*+1/2, *z*−1/2.

### Table 2 Dihedral angles (°) between the mean planes of the aromatic rings

**Table:**

	X-ray	AM1	X-ray	AM1
	Ring-2	Ring-2	Ring-3	Ring-3
Ring-1	59.5 (3)	69.47	44.2 (3)	89.21
Ring-2			24.4 (3)	28.56

Ring-1 is the C1–C6 phenyl ring, Ring-2 is the C15–C20 benzene ring and Ring-3 is the C21–C26 benzene ring.
